# Impact of unanticipated and backhand area smash landing on the lower limb biomechanics of female badminton players

**DOI:** 10.3389/fbioe.2025.1609911

**Published:** 2025-05-30

**Authors:** Zhanyang He, Zeqi Fang, Haoxiang Ye, Sujing Su

**Affiliations:** College of Physical Education and Health Sciences, Zhejiang Normal University, Jinhua, China

**Keywords:** unanticipated, badminton, single leg landing, jump smash, injury risk

## Abstract

**Background:**

In badminton, lower limb injuries frequently occur during unanticipated smash landing movements. Additionally, the risk of lower limb injuries may vary depending on different landing strategies. This study aims to investigate the impact of unanticipated factors and two types of smash actions in the backhand area on lower limb biomechanics.

**Method:**

A motion capture system and force plates were used to collect biomechanic data of 13 female athletes (age: 21.2 ± 1.9 years; height: 167.1 ± 4.1 cm; weight: 57.3 ± 5.1 kg) during backhand rear-court jump smash (BRJS) and backhand lateral jump smash (BLJS) in both anticipated and unanticipated conditions. Unanticipated tasks were conducted by having the athletes perform a random number of specific badminton corner drills, followed by a random movement command given by a signboard and a shuttlecock being launched towards the left half-court by a machine. Waveform analysis was performed using Statistical Parametric Mapping, and discrete parameters were analyzed using a 2 × 2 repeated measures ANOVA.

**Results:**

The results indicated that under unanticipated conditions, both BRJS and BLJS led to higher vertical instantaneous load rates (*p* = 0.003, $\eta_{p}^{2}$ = 0.314) and knee extension moments (*p* = 0.013, $\eta_{p}^{2}$ = 0.231) at initial contact (IC). The main effect results indicated that BRJS caused greater knee abduction angles (*p* = 0.03, $\eta_{p}^{2}$ = 0.182) and knee adduction moments (*p* = 0.010, $\eta_{p}^{2}$ = 0.248) at IC than BLJS, while the interaction effects showed that BRJS had a greater frontal plane center of pressure displacement under unanticipated conditions (*p* = 0.041, $\eta_{p}^{2}$ = 0.186). BLJS showed greater knee extension moments (*p* = 0.013, $\eta_{p}^{2}$ = 0.231) and smaller knee (*p* = 0.002, $\eta_{p}^{2}$ = 0.347) and hip (*p* < 0.001, $\eta_{p}^{2}$ = 0.491) flexion angles at IC compared to BRJS. Additionally, BLJS demonstrated higher peak ankle internal rotation moments (*p* = 0.018, $\eta_{p}^{2}$ = 0.212) than BRJS, with a greater peak ankle inversion moment under unanticipated conditions.

**Conclusion:**

Unanticipated factors significantly impacted the biomechanics of both smash landing actions, potentially increasing the risk of ACL injuries. Moreover, unanticipated factors may increase the risk of ankle sprains during the BLJS movement.

## 1 Introduction

Badminton is a globally practiced sport that involves high-speed, multidirectional movements, making players particularly susceptible to lower limb injuries (Huang et al., 2014). During a match, players must execute a variety of technical movements, including running, jumping, quick stops, and lunges (Shariff et al., 2009). Furthermore, the reactive nature of badminton requires athletes to rapidly respond to opponents’ shots and reposition with minimal delay (Wang et al., 2024). The frequent use of the lower limbs in badminton contributes to a high incidence of lower limb injuries. Epidemiological studies have shown that the incidence of lower limb injuries among professional badminton players ranges from 58.0% to 87.5% (Hensley and Paup, 1979; Jørgensen and Winge, 1987). Anterior cruciate ligament (ACL) injuries and lateral ankle sprains (LAS) are the two most common types of lower limb injuries in badminton (Goh et al., 2013; Kimura et al., 2011; Yung et al., 2007). Smash landing (SL) is the most common attacking movement in badminton (Rambely and Osman, 2005; Sasaki et al., 2018), with the knee and ankle joints being the primary joints for absorbing the impact upon landing (Mills et al., 2009). Excessive ground reaction forces can increase knee loading and ankle instability, thereby increasing the risk of sprains and ACL injuries (Bigouette et al., 2016; Chappell et al., 2005; Norcross et al., 2010).

**FIGURE 1:**
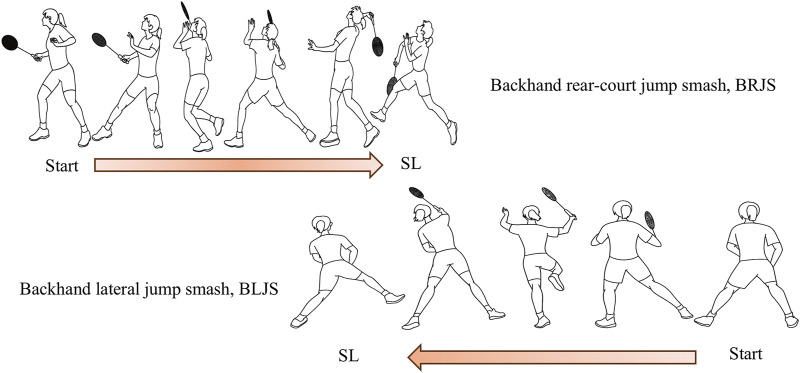
Movement description of BRJS and BLJS.

SL typically involves landing on one leg, with the dominant leg landing in the forehand area and the non-dominant leg landing in the backhand area (Yeow et al., 2011). Badminton is an asymmetrical sport, with significant differences between the dominant and non-dominant sides in terms of bone width, circumference, forearm and thigh girth, and bone mass percentage. These asymmetries can contribute to an increased risk of injury (Abián et al., 2012). Recent epidemiological studies have shown that the incidence of ACL injuries is higher when performing hitting tasks in the backhand side of the rear court, accounting for approximately 19% of all ACL injuries in badminton (Kaldau et al., 2024). Research by Kimura et al. supports this view, finding that the risk of lower limb injury during backhand smash landings (non-dominant leg landing) is higher than that during forehand smash landings (dominant leg landing). Backhand smash landings account for 47.6% of all single-leg landing injuries (Kimura et al., 2010). The backhand rear-court jump smash (BRJS) and backhand lateral jump smash (BLJS) are common backhand jump smash actions in badminton. The BRJS involves side-stepping to an appropriate hitting point, jumping off the dominant leg to execute a smash, and landing on the non-dominant leg (left) while maintaining balance and transferring the body’s center of mass from back to front (using the right hand as an example) (Brahms, 2010; Yu, 2020) (Figure 1). The BLJS requires a quick start to reach the optimal hitting point, followed by a double-leg lateral jump with trunk rotation, flexion, and extension to position for an aerial smash, landing on the non-dominant leg (left) (Yu, 2020) (Figure 1). Both BRJS and BLJS are common movements associated with ACL injuries and LAS in badminton (Fong et al., 2023; Kimura et al., 2010). Furthermore, gender differences play an important role in SL-related injuries. Previous studies have shown that compared to males, females are at a higher risk of LAS and ACL injuries when performing SL in the backhand area due to neuromuscular control and anatomical differences (Hu et al., 2023; Zhao and Gu, 2019). Therefore, the biomechanical mechanisms of non-dominant leg landing injuries in female athletes deserve greater attention (He et al., 2024a).

Most badminton injuries occur during passive-hitting situations, where athletes have limited time to decide on their next actions. This situation may be one of the key factors contributing to injuries. Recent biomechanical studies on lower limb injuries have indicated that factors such as short decision-making time, unanticipated events, and unpredictable movements increase athletes’ cognitive load, thereby raising the risk of lower limb injuries. These factors are crucial elements in injury mechanism research (Jiménez-Martínez et al., 2024). However, previous research on badminton SL has mainly focused on anticipated conditions, examining biomechanical changes related to fatigue (He et al., 2024a; He et al., 2024b; Herbaut and Delannoy, 2020; Rusdiana et al., 2020), gender (Zhao and Gu, 2019), and dominant versus non-dominant limbs (Hu et al., 2023; Zhao and Gu, 2019). Moreover, injury risk assessments have mainly concentrated on closed-skill evaluations of athletic performance, with limited attention given to reactive adaptations to environmental changes (Wilke et al., 2020). In a dynamic sport like badminton, maintaining motor control requires complex integration of constantly changing visual inputs by the central nervous system (Giesche et al., 2021), meaning that focusing only on anticipated conditions is insufficient (Almonroeder et al., 2017; Krosshaug et al., 2006). Additionally, large-scale epidemiological data further support the idea that unanticipated events during rapid visual-motor decision-making processes increase injury risk for athletes (Monfort et al., 2015). Badminton players typically exhibit a choice reaction time of approximately 486.4 ± 99.95 ms (Cheng, 2006). However, during unplanned movements, the available reaction time for altering knee joint mechanics is as short as 300 milliseconds. In such cases, the potential for utilizing neuromuscular feedforward control mechanisms is reduced, thus increasing the risk of related injuries (Almonroeder et al., 2015). This highlights that visual processing speed is crucial for badminton players to successfully execute tactical actions in competition. Athletes must efficiently process complex sensory and visual feedback information within extremely short timeframes (Harpham et al., 2013). To date, there has been a lack of research on the biomechanical relationship between the execution of badminton SL actions and anticipated (visual feedback) factors in injury mechanisms. There is currently no evidence to suggest that anticipated factors are one of the main causes of lower limb injuries in badminton.

Therefore, this study investigates the effects of anticipated factors on lower limb biomechanics during the landing phase of two types of backhand smash landings (BRJS and BLJS) in female badminton players. We hypothesize that, compared to anticipated conditions, unanticipated environments will lead to greater tibial internal rotation moments when badminton players perform smash landings. Additionally, due to differences in movement patterns and landing strategies between the two smash actions (He et al., 2024a), there may be an interaction effect between the smash actions and anticipated factors, leading to different changes in ankle inversion moments for BRJS and BLJS.

## 2 Material and methods

### 2.1 Ethical approval

The experimental protocols and procedures in this study were approved by the Ethics Committee of Zhejiang Normal University, with the Ethical Approval No. ZSRT2023171. Each participant was given a detailed account of the experimental procedures, including the purpose, potential benefits, and possible risks. After that, they provided their written informed consent. It is emphasized that this study was carried out in strict accordance with the principles stipulated in the Declaration of Helsinki.

### 2.2 Participants

The required sample size for this study was determined using G*Power 3.1, employing a significance level (alpha) of 0.05, a target power of 0.80, and an effect size (f) of 0.40, as suggested by He et al. (2024a). Based on these parameters, the calculation indicated that a minimum of 13 participants would be necessary to detect the expected effect (He et al., 2024a). Thirteen female badminton players from universities in Beijing, China, were recruited (age: 21.2 ± 1.9 years; height: 167.1 ± 4.1 cm; weight: 57.3 ± 5.1 kg). All participants voluntarily took part in the study. The inclusion criteria were as follows: Chinese national-ranked badminton players who were right-handed, with no injuries to the trunk, lower limbs, or upper limbs for at least 1 year, and who maintained a training frequency of 4–6 days per week in the month prior to the experiment.

### 2.3 Experimental procedures

The experiment comprised two parts: data collection under anticipated and unanticipated badminton-specific conditions. A senior badminton coach adjusted badminton ball launcher which served the shuttlecock from a designated position based on the players’ hitting height. Prior to the experiment, the badminton ball launcher practiced the serve technique with players, adjusting for standing height, upper limb stretch height, and vertical jump height to ensure serve stability. The shuttlecock serve height was set at 45%–55% of the player’s maximum vertical jump height (measured from the ground with both arms extended overhead), plus the length of the shuttlecock racket’s centerline (Hung et al., 2020). Another coach was present on the sidelines at the start of the experiment to assess the technical quality of the players’ movements (He et al., 2024a).

The experiment was conducted in the Biomechanics Laboratory at Beijing Sport University. Before the study, participants received a detailed explanation of the experimental procedures and subsequently signed informed consent forms. Before the experiment began, participants were asked to warm up on a treadmill at 8 km/h for 6 min, followed by 3 min of static stretching. Oral encouragement was provided before each test to ensure that participants exerted maximum effort. Data collection started with the BRJS task, followed by the BLJS task. After completing each movement, the player returned to the preparation area at the center of the half-court. The unanticipated condition was implemented using a protocol designed to replicate real-time decision-making scenarios. A 20–30 s time interval was maintained between data collections for each task to avoid fatigue interference. Both the coach and players agreed that this interval closely mimicked game speed. Three valid data sets were collected for each task. A task was considered valid if it met the following three criteria: 1) The landing point of the shuttlecock after the jump smash must be within Area A (3.7 × 1.0 m^2^, Figure 1). 2) The off-court coach assessed the quality of the technical movements. 3) The kinematic and dynamic data of the landing movements must be fully collected by infrared high-speed cameras (Vicon) and force platforms.

### 2.4 Unanticipated condition protocol

**FIGURE 2:**
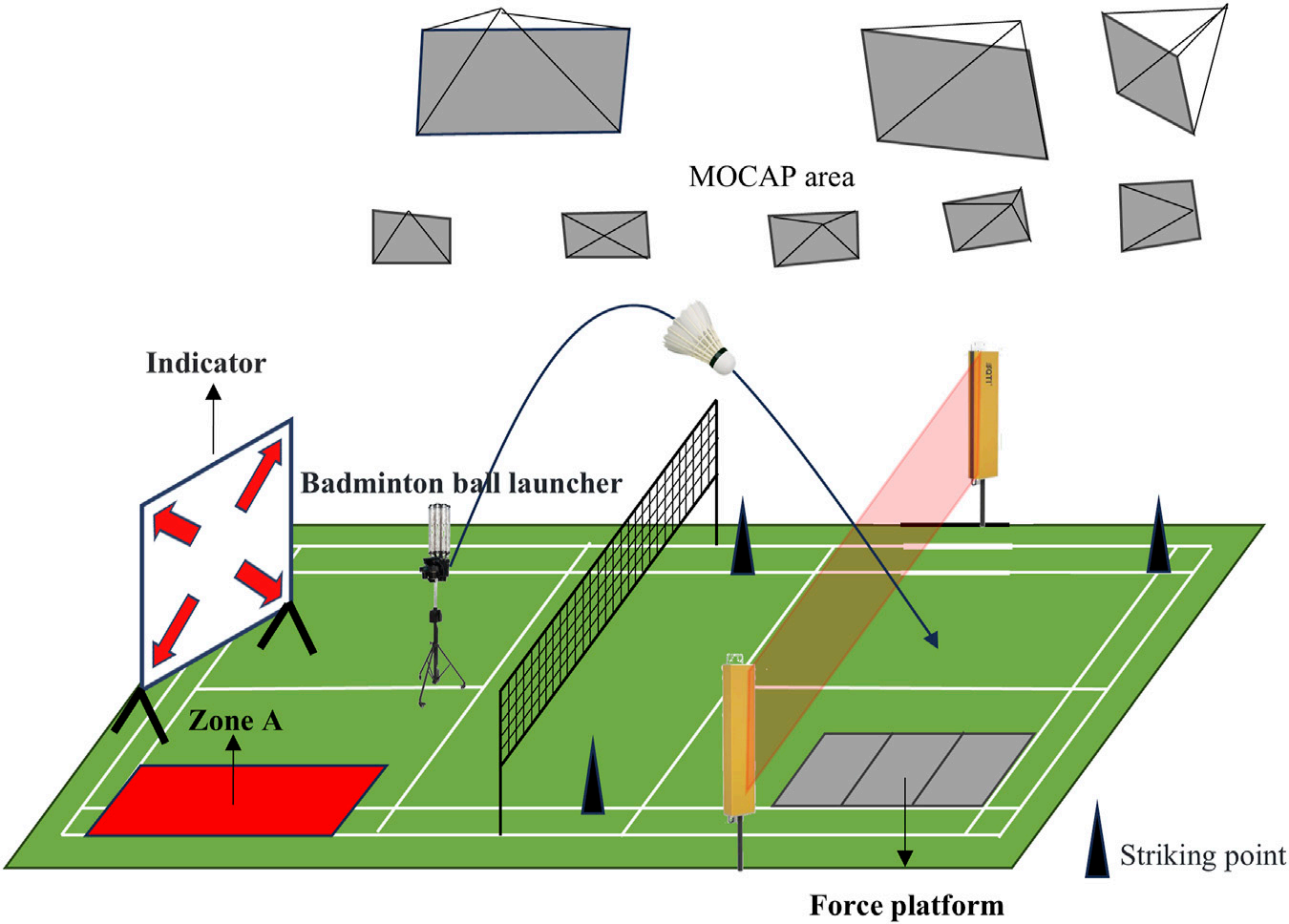
Experimental design description.

Under unanticipated conditions, participants judged the movement direction based on visual cues. Unanticipated conditions were created by modifying the Badminton Specific Speed Test (BST) (Madsen et al., 2015), which increased the randomness of technical movements by combining regular actions with two target actions in four directions: left front, right front, left rear, and right rear. Specifically, the left front and right front directions were assigned to simulate a net shot, while the left rear direction was assigned to two target actions: the BRJS and the BLJS. The right rear direction was assigned to the forehand jump high-clear technique (all regular movements). Markers were placed at each position for hitting (Figure 2).

To more closely mimic game rhythm, participants crossed a turning zone with an infrared barrier for 500 ms, after which they were randomly instructed to move to specific target areas via a display. Participants were required to make a quick decision, move to the target, strike it, and return immediately to the center area for the next round, with all actions performed at maximum speed. During the execution of unanticipated smash landings, after crossing the turning zone with a 300 ms infrared barrier, a random instruction was given by the display, and the opposing court’s serving machine would fire a shuttlecock to the left half-court. The participant was required to quickly strike the shuttlecock into the valid target area. The reason for setting this decision time is that it is the decision time for badminton players in a passive state. To avoid fatigue effects, no more than six movements and hits to the target were allowed during the unanticipated condition trials. Prior to the experiment, a random number between 2 and 6 was selected and entered into a customized system that connected the display to the infrared grid. The grid system then used this number to randomly send commands in three different directions, continuing until the final command for executing either BRJS or BLJS was triggered.

### 2.5 Data collection and processing

**FIGURE 3:**
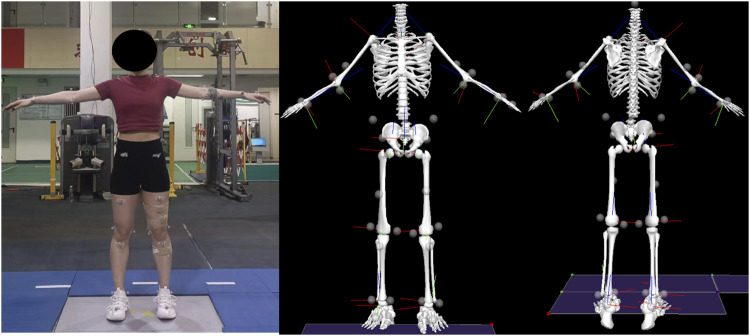
Reflective marker placement diagram.

Eight T40 Vicon cameras (Motion Analysis Raptor-4, United States) with a sampling frequency of 200 Hz were used to capture kinematic data. This equipment was primarily used to collect kinematic parameters of the hip, knee, and ankle joints during the participants’ execution of the jump smash. Three Kistler force platforms (model 9287B, 90 cm × 60 cm × 10 cm, Kistler Instruments AGCorp., Switzerland) were used to measure three-dimensional GRF at a sampling frequency of 1,000 Hz. The motion capture system and force platforms were synchronized. Reflective markers (14 mm diameter) were attached to the participant’s anatomical landmarks using the Helen Hayes marker set, with a total of 24 markers placed on the pelvis, lower limbs, and shoes to capture segmental kinematics (Figure 3) (Kadaba et al., 1990).

The collected data were analyzed using Visual3D software (v6, c-motion, inc., Rockville, MD). Marker and GRF data were filtered using a fourth-order Butterworth filter with a cutoff frequency of 10 Hz. The 3D angles of the hip and knee joints were calculated using the Cardan-Euler method and were transformed into the joint coordinate system (Davis III et al., 1991). All movement data were normalized by body weight (BW). The initial contact (IC) during landing was determined by identifying the first frame where the force exceeded 10 N, and the analysis range extended from the moment of contact with the ground to the maximum knee flexion angle.

### 2.6 Research variables

Based on the ACL injury mechanism during landing, the variables related to ACL injury in this study were defined as: kinematic and dynamic parameters of the hip, knee, and ankle joints at the IC moment; knee joint moment at the first peak vertical ground reaction force (VGRF) moment; joint range of motion (ROM) for the hip, knee, and ankle (calculated as the difference between the maximum and minimum angles in a single plane); lower limb vertical instantaneous load rates (VILR) [VILR = Max (ΔvGRF/Δt)] and vertical average load rates (VALR) (VALR = (VGRF _peak vGRF_–VGRF _IC_)/(t _peak vGRF_–t _IC_) (Zhu et al., 2024).

For the LAS injury mechanism, the variables related to LAS injury were defined as: ankle joint angle during the landing phase; ankle joint peak moment; and the maximum deviation of the center of pressure (COP) in the sagittal and frontal planes during the phase (DT et al., 2009).

To further explore the impact of the two factors (Anticipated × Movement) on lower limb biomechanics, random vector field theory was used to explain the variability of data during the landing phase, including vertical ground reaction force (VGRF), joint angles (hip, knee, and ankle), and moments.

### 2.7 Statistical analysis

The significance level for all statistical tests was set at 0.05, with data presented as mean ± standard deviation. The normality of each variable was tested using the Shapiro–Wilk test, which showed that the experimental data followed a normal distribution. Statistical analyses were performed using SPSS 17.0 (SPSS Inc., Chicago, IL, United States). A 2 × 2 repeated measures ANOVA (jump smash movement × expected condition) was used to analyze the effects of the jump smash movement and expected conditions on lower limb biomechanics. When the main effects were significant, *post hoc* comparisons were conducted using paired t-tests with Bonferroni correction. Open-source software package spm1d (http://spm1d.org) in MATLAB R2019a (The MathWorks, Natick, MA, United States) was used for analysis (Pataky, 2010). Statistical parameter mapping (SPM) and 2 × 2 repeated measures ANOVA were applied to analyze the time series data of VGRF, joint angles, and moments. Partial eta squared was used as the effect size, with the following interpretations: 0.01 for a small effect, 0.06 for a medium effect, and 0.14 for a large effect (Vacha-Haase and Thompson, 2004).

## 3 Result

### 3.1 Kinematics

The kinematic analysis indicated that, compared to the anticipated condition, the unanticipated condition led to a significant increase in ankle eversion/inversion ROM [F (1,38) = 12.58, *p* = 0.039, $\eta_{p}^{2}$ = 0.344] and hip internal/external rotation ROM [F (1,38) = 9.65, *p* = 0.001, $\eta_{p}^{2}$ = 0.287]. The main effects of the movement were significant for several key kinematic parameters: maximum displacement of the COP in the sagittal plane [F (1,38) = 5.91, *p* = 0.023, $\eta_{p}^{2}$ = 0.198], ankle dorsiflexion/plantarflexion ROM [F (1,38) = 10.385, *p* = 0.004, $\eta_{p}^{2}$ = 0.302], ankle internal/external rotation ROM [F (1,38) = 10.64, *p* = 0.003, $\eta_{p}^{2}$ = 0.307], hip flexion/extension ROM [F (1,38) = 24.042, *p* < 0.001, $\eta_{p}^{2}$ = 0.5], hip internal/external rotation ROM [F (1,38) = 10.524, *p* = 0.003, $\eta_{p}^{2}$ = 0.305], knee flexion [F (1,38) = 12.417, *p* = 0.002, $\eta_{p}^{2}$ = 0.347], abduction [F (1,38) = 5.347, *p* = 0.03, $\eta_{p}^{2}$ = 0.182], and external rotation angles [F (1,38) = 4.321, *p* = 0.049, $\eta_{p}^{2}$ = 0.153] at IC, as well as the hip flexion angle [F (1,38) = 23.116, *p* < 0.001, $\eta_{p}^{2}$ = 0.491] (Supplementary Table S1). In comparison to BRJS, BLJS showed a smaller maximum displacement of COP in the sagittal plane, smaller ankle dorsiflexion/plantarflexion ROM, smaller ankle internal/external rotation ROM, and smaller hip flexion/extension and internal/external rotation ROM. At the landing moment, BLJS exhibited a smaller knee flexion angle, a smaller knee abduction angle, a larger knee external rotation angle, and a smaller hip flexion angle (Table 1).

**TABLE 1:** Comparison of the mean and standard deviation of Kinematic parameters for BRJS and BLJS Anticipated and Unanticipated.

Variable		BRJS		BLJS		*p* -value		
		Anti	Un-anti	Anti	Un-anti	Anti	Movement	Interaction
COP (mm)								
The Sagittal plane displacement		169.78 ± 29.06^a^	169.73 ± 25.53	132.86 ± 34.11^a^	158.98 ± 46.85	0.184	**0.023**	0.182
The Front plane displacement		45.48 ± 16.19^b^	59.35 ± 20.85^a,b^	41.47 ± 13.18	37.57 ± 18.98^a^	0.239	**0.028**	**0.041**
ROM (°)								
Ankle	Extension-Flexion	45.33 ± 6.42^a^	45.21 ± 3.44^a^	38.92 ± 5.01^a^	41.28 ± 4.58^a^	0.324	**0.004**	0.276
	Eversion-Inversion	6.84 ± 4.34^b^	8.93 ± 5.04^b^	5.62 ± 3.59^b^	8.97 ± 5.6^b^	**0.039**	0.666	0.618
	External-Internal Rot	8.34 ± 3.89^a^	9.78 ± 2.6^a^	5.12 ± 3.57^a^	6.41 ± 3.07^a^	0.107	**0.003**	0.929
Knee	Extension-Flexion	28.69 ± 9.86	30.3 ± 7	32.878 ± 7.37	33.16 ± 9.12	0.66	0.177	0.758
	Abduction-Adduction	3.28 ± 2.03	4 ± 2.21^a,b^	4.18 ± 2.36	4.42 ± 3.03^a^	0.478	0.347	0.719
	External-Internal Rot	5.68 ± 3.90	5.13 ± 1.89	4.48 ± 2.22	5.21 ± 2.25	0.85	0.553	0.211
Hip	Extension-Flexion	39.76 ± 11.91^a^	41.54 ± 12.62^a^	25.85 ± 9.41^a^	25.53 ± 7.32^a^	0.797	**<0.001**	0.709
	Abduction-Adduction	11.12 ± 5.98	15.43 ± 5.32^b^	11.39 ± 4.91	12.44 ± 5.49	0.097	0.362	0.305
	External-Internal Rot	11.94 ± 5.74^a,b^	18.83 ± 7.31^a,b^	7.24 ± 5.72^a,b^	10.96 ± 4.82^a,b^	**0.001**	**0.003**	0.238
Joint angle at IC (°)								
Ankle	Dorsiflexion (+)	−21.97 ± 4.64^a^	−19.98 ± 3.91	−18.98 ± 3.8^a^	−18.88 ± 3.65	0.34	0.088	0.389
	Eversion (+)	11.59 ± 7.07	14.15 ± 4.07	12.88 ± 6.15	16.47 ± 5.11	0.086	0.223	0.765
	External Rot (+)	0.91 ± 6.99	−0.55 ± 6.17	3.39 ± 5.59	2.55 ± 6.29	0.366	0.202	0.804
Knee	Extension (+)	−24.17 ± 5.95^a^	−23.11 ± 6.90^a^	−18.00 ± 5.28^a^	−16.34 ± 4.46^a^	0.303	**0.002**	0.818
	Abduction (+)	2.06 ± 1.82	2.64 ± 1.99	1.20 ± 1.69	1.44 ± 1.67	0.463	**0.03**	0.753
	External Rot (+)	9.81 ± 4.03	9.37 ± 3.25^a^	11.66 ± 3.43	12.7 ± 4.32^a^	0.715	**0.049**	0.373
Hip	Flexsion (+)	30.71 ± 6.59^a^	29.17 ± 10.25^a^	16.99 ± 6.46^a^	16.75 ± 12.94^a^	0.727	**<0.001**	0.799
	Abduction (+)	42.43 ± 4.47^a^	40.56 ± 6.17	37.13 ± 2.75^a,b^	40.93 ± 2.45^b^	0.367	0.067	**0.013**
	External Rot (+)	−4.94 ± 10.34	−1.87 ± 14.24	−4.71 ± 9.02	−1.05 ± 10.13	0.167	0.887	0.903

^a^Statistically significant difference compared with other action (<0.05).

^b^Statistically significant difference between Anticipated and Unanticipated (<0.05).

The bold value is p-value<0.05.

The interaction between expected conditions and movement type significantly influenced the maximum displacement of COP in the frontal plane [F (1,38) = 5.487, *p* = 0.041, $\eta_{p}^{2}$ = 0.186] and hip abduction [F (1,38) = 2.56, *p* = 0.050, $\eta_{p}^{2}$ = 0.096] at IC. Post hoc tests revealed that under unanticipated conditions, the maximum COP displacement in the frontal plane significantly increased for BRJS (*post hoc* < 0.05). Under unanticipated conditions, BLJS also showed a significant decrease in hip abduction at IC (*post hoc* <0.05).

**FIGURE 4:**
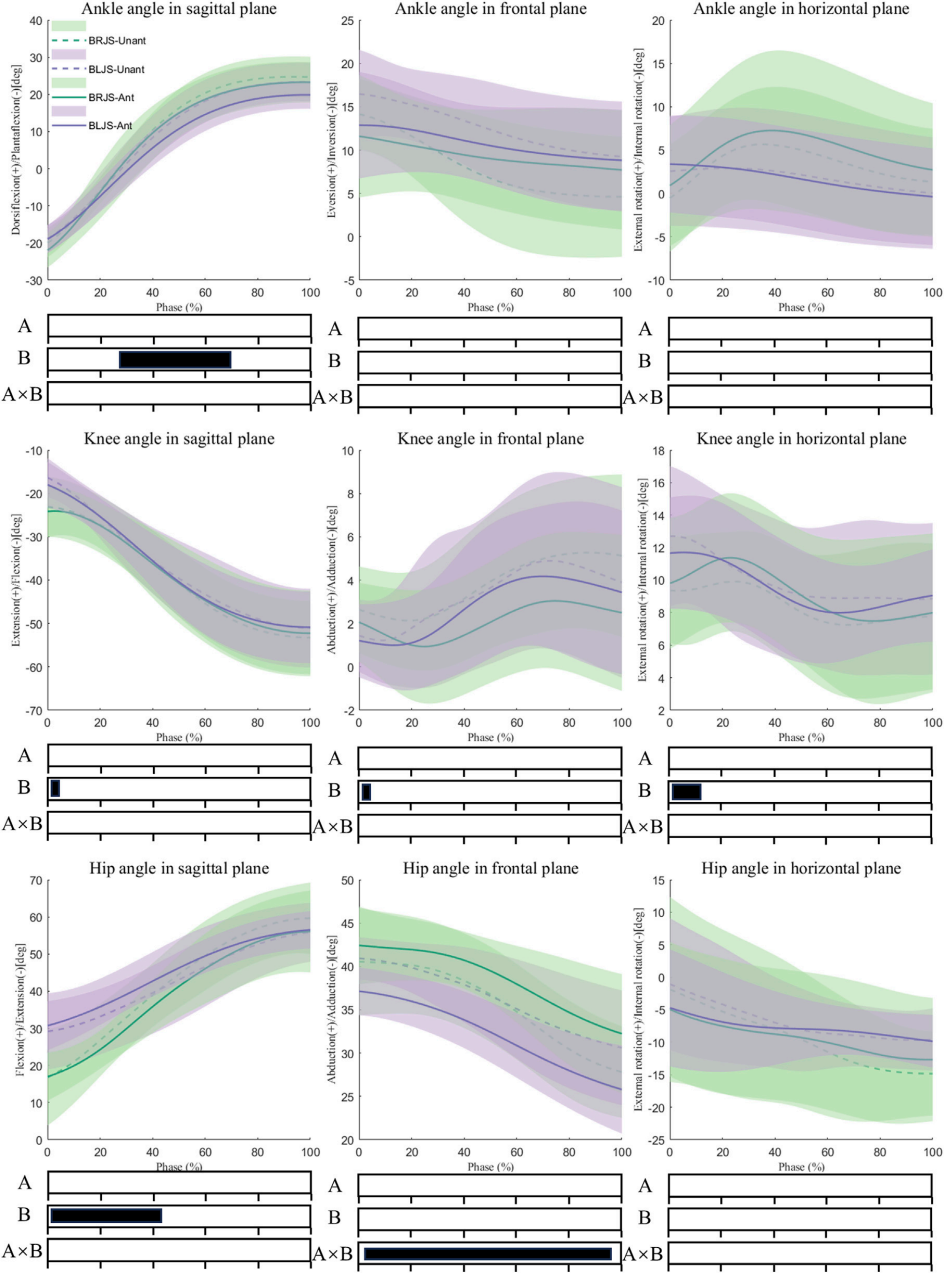
Lower limb kinematics during the SL phase. Ant: Anticipated; Unant: Unanticipated; A: Movement Effect; B: Anticipated Effect; A × B: Interaction Effect between Movement and Anticipated.

Multivariate vector field analysis using spm1d revealed an interaction effect on the hip frontal angle from 0% to 100% during the landing phase (*P* < 0.001). Post hoc tests indicated that BLJS, under unanticipated conditions, exhibited a smaller hip abduction angle. For the smash movements, significant changes were observed in the ankle dorsiflexion angle from 34.1% to 67.3% during the landing phase (*p* = 0.024). Additionally, significant differences were noted in the knee flexion angle from 0% to 2.5% (*p* = 0.050), knee abduction angle from 0% to 4.9% (*p* = 0.050), knee external rotation angle from 0% to 8.8% (*p* = 0.048), and hip flexion angle from 0% to 42.4% (*p* = 0.019). BRJS exhibited larger knee flexion angles, and smaller ankle dorsiflexion angles, knee internal rotation angles, and hip flexion angles (Figure 4). The summary of the significance of SPM is located in Supplementary Table S2.

### 3.2 Kinetics

Kinetic analysis revealed that, under the unanticipated condition, VILR [F (1,38) = 10.988, *p* = 0.003, $\eta_{p}^{2}$ = 0.314], knee extension moment [F (1,38) = 7.191, *p* = 0.013, $\eta_{p}^{2}$ = 0.231] at IC, and hip abduction moment [F (1,38) = 6.69, *p* = 0.016, $\eta_{p}^{2}$ = 0.218] at IC were significantly greater than under the anticipated condition. Compared to BRJS, BLJS exhibited a smaller ankle internal rotation moment at IC [F (1,38) = 12.613, *p* = 0.002, $\eta_{p}^{2}$ = 0.344], larger knee extension moment at IC [F (1,38) = 4.59, *p* = 0.043, $\eta_{p}^{2}$ = 0.161], smaller knee adduction moment at IC [F (1,38) = 7.921, *p* = 0.01, $\eta_{p}^{2}$ = 0.248], smaller knee external rotation moment at IC [F (1,38) = 5.537, *p* = 0.027, $\eta_{p}^{2}$ = 0.187], larger hip extension moment at IC [F (1,38) = 7.517, *p* = 0.011, $\eta_{p}^{2}$ = 0.238], larger hip internal rotation moment at IC [F (1,38) = 12.458, *p* = 0.002, $\eta_{p}^{2}$ = 0.342], and a smaller peak ankle internal rotation moment [F (1,38) = 9.305, *p* = 0.005, $\eta_{p}^{2}$ = 0.053]. The ankle inversion-eversion peak moment showed an interaction effect [F (1,38) = 6.455, *p* = 0.018, $\eta_{p}^{2}$ = 0.212], with *post hoc* tests revealing a significant increase in eversion angle for BLJS under the unanticipated condition compared to the anticipated condition (Table 2).

**TABLE 2:** Comparison of the mean and standard deviation of kinetic parameters for BRJS and BLJS Anticipated and Unanticipated.

Variable		BRJS		BLJS		P-value		
		Anti	Un-anti	Anti	Un-anti	Anti	Movement	Interaction
loading rate	VALR	37.08 ± 5.72	44.89 ± 11.58	38.92 ± 6.91	44.26 ± 12.48	0.052	0.762	0.703
	VILR	83.52 ± 24.81^b^	114.29 ± 38.12^b^	97.39 ± 18.61^b^	124.34 ± 48.33^b^	**0.003**	0.259	0.828
Joint moment at IC (Nm/kg)								
Ankle	Dorsiflexion (+)	−0.43 ± 0.07	−0.44 ± 0.07	−0.48 ± 0.08	−0.46 ± 0.10	0.698	0.198	0.435
	Eversion (+)	−0.03 ± 0.07	−0.01 ± 0.06	0.02 ± 0.07	0 ± 0.08	0.88	0.207	0.091
	External Rot (+)	−0.11 ± 0.04^a^	−0.11 ± 0.03^a^	−0.056 ± 0.05^a^	−0.07 ± 0.04^a^	0.718	**0.002**	0.406
Knee	Extension (+)	0.02 ± 0.21^a,b^	0.19 ± 0.18^b^	0.19 ± 0.19^a^	0.27 ± 0.17	**0.013**	**0.043**	0.362
	Abduction (+)	−0.57 ± 0.12^a^	−0.53 ± 0.10^a^	−0.44 ± 0.15^a^	−0.42 ± 0.14^a^	0.166	**0.01**	0.691
	External Rot (+)	0.18 ± 0.05^a^	0.18 ± 0.073^a^	0.14 ± 0.07^a^	0.13 ± 0.07^a^	0.639	**0.027**	0.661
Hip	Flexsion (+)	0.19 ± 0.16^a^	0.23 ± 0.30	−0.09 ± 0.39^a^	0.05 ± 0.32	0.299	0.011	0.554
	Abduction (+)	−1.09 ± 0.20^b^	−0.94 ± 0.16^b^	−0.91 ± 0.25	−0.87 ± 0.22	**0.016**	0.11	0.207
	External Rot (+)	−0.17 ± 0.09^a^	−0.19 ± 0.17^a^	−0.32 ± 0.1^a^	−0.31 ± 0.11^a^	0.718	**0.002**	0.554
Joint moment at 1st vGRF phase								
Knee	Extension (+)	0.32 ± 0.22	0.44 ± 0.28	0.33 ± 0.24^b^	0.64 ± 0.3^b^	**0.005**	0.178	0.188
	Abduction (+)	−0.75 ± 0.2^a^	−0.71 ± 0.16^a^	−0.47 ± 0.23^a^	−0.52 ± 0.18^a^	0.918	**0.001**	0.301
	External Rot (+)	0.21 ± 0.06^a^	0.21 ± 0.11	0.14 ± 0.08^a^	0.18 ± 0.11	0.285	0.077	0.28
Peak ankle moment (Nm/kg)								
Dorsiflexion (+)		−1.86 ± 0.36	−1.68 ± 0.23	−1.78 ± 0.30	−1.79 ± 0.21	0.276	0.875	0.226
Eversion (+)		−0.07 ± 0.13	−0.05 ± 0.1	−0.01 ± 0.12^b^	−0.09 ± 0.13^b^	0.109	0.733	**0.018**
External Rot (+)		−0.27 ± 0.14^a^	−0.22 ± 0.09^a^	−0.13 ± 0.11^a^	−0.13 ± 0.09^a^	0.26	**0.005**	0.189

^a^Statistically significant difference compared with other action (<0.05).

^b^Statistically significant difference between Anticipated and Unanticipated (<0.05).

The bold value is p-value<0.05.

**FIGURE 5:**
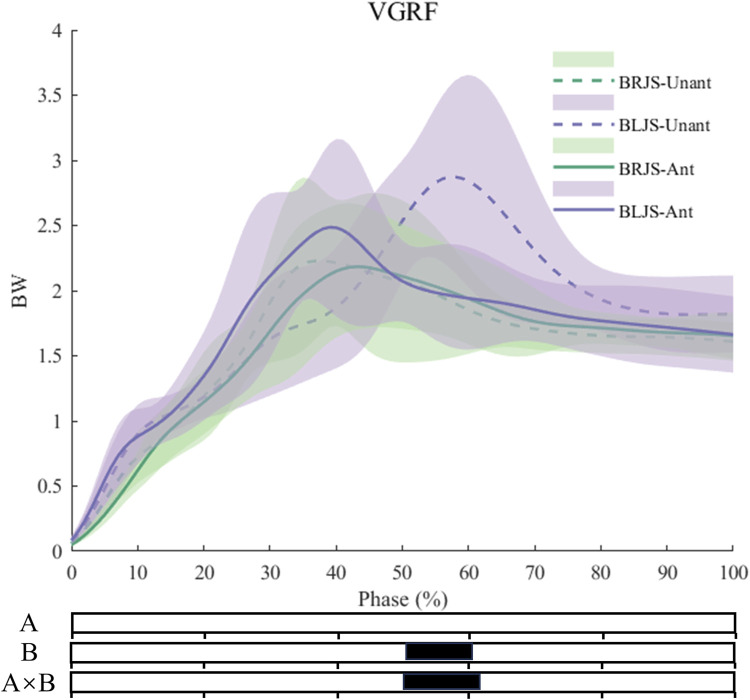
VGRF during the SL phase. Ant: Anticipated; Unant: Unanticipated; A: Movement Effect; B: Anticipated Effect; A × B: Interaction Effect between Movement and Anticipated.

**FIGURE 6:**
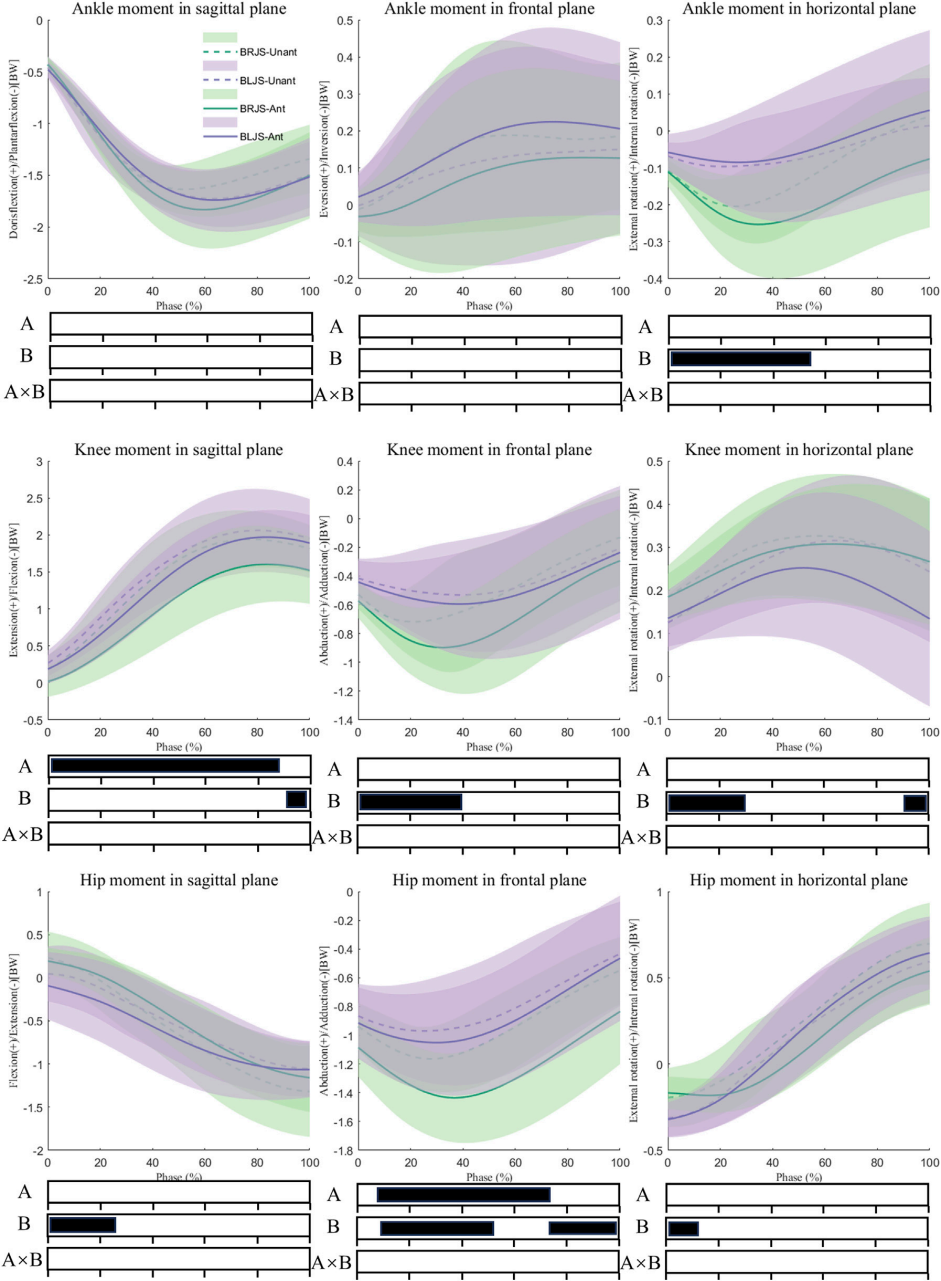
Lower limb moment during the SL phase. Ant: Anticipated; Unant: Unanticipated; A: Movement Effect; B: Anticipated Effect; A × B: Interaction Effect between Movement and Anticipated.

Multivariate vector field analysis using spm1d revealed interaction effects in VGRF during the 53.1%–62.1% phase of the landing (*p* = 0.003) (Figure 5), with *post hoc* tests showing larger VGRF for BLJS under the unanticipated condition. Under the main effect of the unanticipated condition, a greater knee extension moment (*p* < 0.001) was observed during the 0%–86.6% phase of landing, as well as a smaller hip adduction moment during the 9.8%–87.5% phase of landing (*p* = 0.004). Compared to BRJS, BLJS exhibited a smaller ankle internal rotation moment during the 0%–64.2% phase of landing (*p* = 0.003), a larger knee extension moment during the 86.4%–100% phase of landing (*p* = 0.044), smaller knee abduction moment during the 0%–40.9% phase of landing (*p* = 0.014), and smaller knee external rotation moment during the 0%–32.2% and 91%–100% phases of landing (*p* = 0.03, *p* = 0.048). BLJS also showed a larger hip extension moment during the 0%–26.6% phase of landing (*p* = 0.035) and a smaller hip adduction moment during the 10.2%–51.5% and 72.3%–100% phases of landing (*p* = 0.024, *p* = 0.036). Additionally, BLJS exhibited a larger hip internal rotation moment during the 0%–10.6% phase of landing (*p* = 0.045) (Figure 6).

## 4 Discussion

Current biomechanics research on unexpected factors primarily focuses on laboratory experiments involving landing, cutting, and similar movements (Niemeyer et al., 2019; Zhu et al., 2024), with relatively limited evidence strength and weak applicability to real-world sport-specific contexts. Additionally, biomechanical studies investigating the injury mechanisms of specific movements under expected conditions remain relatively scarce (Giesche et al., 2021). This study aimed to simulate factors scheduled within the context of badminton to explore the lower limb biomechanical differences between two SL actions in badminton.

Our results did not support the first hypothesis of this study, as no significant statistical differences were found in knee torsional moments throughout the entire SL phase. However, the second hypothesis was supported, as our study revealed an interaction effect on ankle inversion-eversion peak moments. Post hoc tests showed that BLJS under unanticipated conditions exhibited larger ankle inversion moments, whereas BRJS was unaffected by the unanticipated factors.

### 4.1 Effects of anticipated factors on SL

This study found that under unexpected conditions, female badminton players performing BRJS and BLJS exhibited increased ankle frontal ROM, increased hip transverse plane ROM, higher VILR, increased knee extension moments at IC, decreased hip adduction moments at IC, and increased knee extension moments at the first peak of VGRF. SPM analysis confirmed a sustained increase in knee extension moments during the 0%–86.6% phase of landing and decreased hip adduction moments during the 9.8%–87.5% phase during landing. These findings contrast with those of Giesche et al. (2021), who conducted a combined analysis of lower limb biomechanics during running or single-leg landing-cutting actions under expected and unexpected conditions. They found larger knee abduction moments and greater tibial internal rotation moments under unanticipated conditions. However, no significant differences were observed in the sagittal plane (Giesche et al., 2021). The discrepancy may be because their review mainly focused on single-leg landing-cutting actions, while our study exclusively examined SL movements under unexpected conditions. Moreover, athletes typically make two to three quick steps of adjustment before executing the SL after receiving instructions, suggesting differences in movement patterns and feedforward mechanisms involving motor planning and cognition (McLean et al., 2010; Shultz et al., 2003; Swanik et al., 2006). Additionally, our experimental design required athletes to continuously track changing environmental factors, such as the positions of the players and the shuttlecock, in their short-term visual memory. This ability involves feedforward movement planning, which differs significantly from previous experiments that relied solely on time-limited visual decision-making tasks (Smith and Mitroff, 2012). Overall, the biomechanical differences observed in this study under unexpected conditions can be attributed to changes in movement patterns and the athletes’ feedforward movement planning.

We observed that under unexpected conditions, athletes executing BRJS and BLJS exhibited higher VILR, which is often associated with greater knee extension moments (Blackburn and Padua, 2007). Larger knee extension moments were observed at both IC and the first peak of VGRF, and SPM results also indicated that this effect persisted across a larger time window (0%–86.6%). A substantial body of prospective research suggests that the most common non-contact injury mechanisms in female athletes occur during deceleration tasks involving greater knee extension moments, regardless of visual interference (Carlson et al., 2016; Krosshaug et al., 2006; Olsen et al., 2004). Laboratory-based studies have supported this conclusion, showing that ACL load is positively correlated with knee extension moments and that isolated quadriceps contraction can lead to ACL injuries or tears (DeMorat et al., 2004; Dürselen et al., 1995; Li et al., 1999). In this process, the combined effect of higher VILR and increased knee extension moments contributes to increased sagittal shear force on the tibial plateau, thereby amplifying ACL stress and ultimately raising the injury risk for both movements. Additionally, we observed a reduction in hip adduction moments at IC and during the 9.8%–87.5% phase of landing. Previous research has identified excessive hip adduction moments as a significant contributor to increased knee abduction moments (Ireland, 2003; Powers, 2003; Willson et al., 2005), with larger knee abduction moments being a known mechanism for ACL injury in the frontal plane (Hewett et al., 2005; Markolf et al., 1995). Surprisingly, this study revealed a decrease in hip adduction moments under unexpected conditions during SL execution, and no significant changes in knee abduction moments were observed. These findings imply that ACL injury mechanisms in BRJS and BLJS are likely dominated by sagittal-plane shear forces rather than frontal-plane dynamics (Hewett et al., 2010).

Furthermore, this study found that, under unexpected conditions, athletes performing BRJS and BLJS exhibited greater hip internal-external rotation ROM and ankle inversion-eversion ROM. During landing and cushioning, larger joint ROM in the horizontal and transverse planes helps effectively dissipate the ground reaction forces in the sagittal and transverse planes (Smith et al., 2009). This increased ROM may reflect a compensatory strategy to stabilize the lower limb in response to unanticipated landings. Previous studies have found a positive correlation between increased ankle eversion ROM and a reduced risk of lower limb injury (Hoch et al., 2015; Padua et al., 2019), as it helps reduce the impact forces on the foot during landing, subsequently decreasing the transmitted forces on the knee, hip, and trunk. Additionally, hip internal-external rotation ROM plays an important role in reducing knee joint stress, particularly during unanticipated landings, where hip rotation adjustments are considered an effective protective mechanism. Previous research has shown that smaller hip ROM is significantly correlated with greater knee abduction motion (*R* ^2^ = 0.475, P < 0.01), suggesting that larger hip ROM can effectively reduce the external load on the knee joint (Uota et al., 2017). These protective mechanisms differ significantly from those identified by Giesche et al. (2021). In their meta-analysis of 14 studies on cutting movements, Giesche et al. found that athletes performing cutting movements under unexpected conditions generated larger knee abduction and tibial internal rotation moments, which effectively increased the internal stress on the knee joint. The reason for the discrepancy with the previous review results may lie in the fact that their study focused on cutting tasks, while this study focused on landing tasks, which differ fundamentally in terms of movement patterns (Giesche et al., 2021).

In unexpected environments, athletes performing BRJS and BLJS generate greater knee extension moments and VILR, resulting in increased shear forces on the ACL in the sagittal plane, thereby raising the risk of ACL injury. However, the protective mechanisms involving increased hip internal-external rotation ROM and ankle inversion-eversion ROM appear to reduce internal stress on the knee joint. A study analyzing video footage of ACL injuries in 39 basketball players found that ACL injuries often occur when the knee flexion angle is between 20 and 30° after landing (Giesche et al., 2021), which corresponds to the 0%–30% phase of SL landing in this study. This phase represents a high-risk range for ACL injuries. This study observed that under unexpected conditions, VILR occurred during the 10%–20% phase of SL landing (Gruber et al., 2013), while larger knee extension moments persisted throughout the 0%–86.6% phase of SL landing. Therefore, the overlap between higher VILR, knee extension moments, and the ACL injury exposure range during SL landing significantly increases the risk of injury. Furthermore, the protective mechanisms identified in this study seem to become effective only after 86.6% of the SL phase, when the knee extension moment begins to decrease.

### 4.2 Effect of movement factors on SL

The second objective of this study was to explore the biomechanical differences in lower limb landing during backhand serve SL actions between BRJS and BLJS, and how these differences affect LAS and ACL injury mechanisms. Due to the different landing directions, participants adopt distinct lower limb landing strategies, leading to variations in biomechanical parameters (Sinsurin et al., 2017).

Compared to BRJS, BLJS demonstrated smaller hip flexion and knee flexion angles during the IC phase, alongside larger hip extension and hip internal rotation moments. The lateral landing type, specifically the sideward external landing, exhibited a smaller hip flexion angle at IC, a phenomenon supported by multiple biomechanical studies on unilateral landing in different directions (Lee and Lee, 2012; Sinsurin et al., 2017). Additionally, research by Sinsurin et al. examined the biomechanical differences in volleyball players’ single-leg landings in forward, lateral (30° and 60°), and sideward (90°) directions (Sinsurin et al., 2017). They found that, compared to other landing directions, the sideward single-leg landing showed a larger knee extension moment at peak vertical GRF, which aligns with our findings (Sinsurin et al., 2017). However, their study also found no significant differences in knee flexion angles compared to other landing directions, which contrasts with our results (Sinsurin et al., 2017). He et al. also observed similar discrepancies when comparing ankle joint biomechanical parameters during SL landings in badminton players under standard laboratory conditions (He et al., 2024a). This discrepancy may be attributed to the differing movement patterns between badminton SL actions and laboratory-based single-leg landing measurements.

Previous prospective studies on ACL injury mechanisms have pointed out that a more upright body posture resulting from smaller knee and hip flexion angles during landing increases the load on the ACL, thereby raising the risk of ACL injury (Griffin et al., 2000). Additionally, smaller knee and hip flexion angles during landing, combined with higher quadriceps strength and lower hamstring strength (resulting in an increased knee extension moment), may contribute to the sagittal plane mechanism of ACL injury (He et al., 2024a). In such cases, a larger angle between the patellar tendon and tibial vertical axis increases forward knee shear load, thereby further raising the risk of ACL injury (Pandy and Shelburne, 1997).

Interestingly, both BRJS and BLJS appear to present a risk for ACL injury; however, their underlying biomechanical mechanisms for potential injury differ. Compared to BLJS, BRJS shows larger knee abduction angles, greater knee internal rotation moments, and smaller hip internal rotation moments at IC, along with an increase in knee internal rotation moments at the first peak of GRF. The results from SPM analysis support these findings. Previous studies explain this phenomenon: smaller hip internal rotation moments reduce hip internal rotation stability, leading to difficulty in maintaining proper knee alignment during landing, which increases knee abduction angles (Lucas et al., 2017). Moreover, previous prospective and case-control studies have identified larger knee abduction angles and greater knee adduction moments as predictors of ACL injuries (Chou et al., 2023; Koga et al., 2010; Quatman and Hewett, 2009). Larger knee internal rotation moments increase pressure on the knee’s medial side, significantly amplifying shear forces on the ACL (Hewett et al., 2005). Specifically, during landing, an increase in knee internal rotation moment heightens the risk of ACL injury. Additionally, when the knee abduction angle during landing becomes excessively large, knee stability is significantly compromised, resulting in increased stress on the ACL and a higher likelihood of injury.

This study also observed significant differences in ankle internal rotation (peak moment) between SPM and 0D analyses, with BLJS showing higher peak ankle internal rotation moments. Sinsurin et al. observed that lateral landings produce greater ankle internal rotation moments compared to other landing directions, a finding confirmed by He et al. (2024a), Sinsurin et al. (2017). Lateral single-leg landings increase the lateral component of the GRF, requiring the ankle joint to counteract this force by generating higher internal rotation moments. Previous studies have demonstrated that large internal rotation moments during the landing phase can trigger explosive ankle retroversion or inversion movements (Pataky, 2010), thus increasing the risk of LAS.

### 4.3 Interaction between smash movements and anticipatory factors

BRJS and BLJS are common offensive actions in the backhand area (Rambely and Osman, 2005; Sasaki et al., 2018), and they are partially interchangeable in backhand smash (He et al., 2024a). In situations where athletes encounter unexpected conditions and limited decision-making time, selecting a relatively safe smash movement becomes crucial. Our results reveal interactions between the frontal plane COP, peak ankle inversion moment, hip abduction angle at IC, and GRF under SPM. Post-hoc tests show that BRJS leads to a significantly larger frontal plane COP under unexpected conditions, whereas BLJS shows no significant differences in anticipatory factors. For BLJS, peak ankle inversion moment, hip abduction angle at IC, and GRF under SPM significantly increase in unexpected conditions. We find that the simple effects of anticipatory factors post-interaction differ across different landing modes (lateral landing and rearward landing). Previous studies suggest that visual-motor function and decision response time may affect neuromuscular function and postural control abilities (Harpham et al., 2013; Mihalik et al., 2010). Athletes perform BRJS with longer displacement than BLJS, leading to differences in movement patterns and decision response times between the two actions. When neuromuscular function and postural control ability decrease due to limited decision-making time, variations in biomechanical parameters arise across different movement modes. This biomechanical phenomenon has been observed in fatigue-induced reductions in neuromuscular function (Gholipour Aghdam et al., 2024).

COP is an important indicator for assessing an athlete’s postural control during SL actions (Sung et al., 2020). Our study found that BRJS under unexpected conditions significantly increases frontal plane COP, indicating a decrease in postural control ability compared to expected conditions. Lin et al.’s research supports our finding that increased COP displacement under limited decision-making time is a self-protection mechanism of athletes (Lin et al., 2022). In unexpected environments, the body must react more rapidly, and during this process, the pre-activation (and feedforward response) of muscles is reduced (Giesche et al., 2021), necessitating a larger COP trajectory to make more dynamic adjustments and maintain balance, thus preventing falls or injuries (Lin et al., 2022). Furthermore, BRJS’s larger frontal plane COP displacement under unexpected conditions leads to an outward shift in the GRF vector, increasing knee abduction movement (Bendjaballah et al., 1997; Hewett et al., 2005). Upon comparing the main effects, we found that BRJS carries a higher risk of frontal plane ACL injury mechanisms, as it results in larger knee abduction angles and greater knee internal rotation moments compared to BLJS. The larger frontal plane COP displacement during BRJS under unexpected conditions indirectly increases the knee abduction moment arm, thus raising the risk of ACL injury.

In the interaction effects, BLJS shows higher peak ankle inversion moment, larger hip abduction angles at IC and across the 0%–100% phase (SPM results), and larger VGRF within the SL phase range of 53.1%–62.1% under unexpected conditions. Previous studies examining the impact of cutting maneuvers on lower limb joints and muscles under unexpected conditions also found increased hip abduction angles, suggesting that female athletes tend to increase hip abduction during unexpected changes in direction. This adjustment likely occurs to enhance lower limb stability and prevent injury to the knee and ankle joints during sudden movements (Pollard et al., 2004). Although BLJS may exhibit smaller hip abduction angles under unexpected conditions to maintain lower limb stability, it inevitably results in larger peak ankle inversion moment and greater VGRF, which may contribute to biomechanical variables leading to lower limb injuries. Prior research has shown that a significant number of LAS events are caused by explosive retroversion or inversion moments at the ankle (DT et al., 2009). Therefore, executing BLJS under limited decision-making time increases the risk of LAS. Furthermore, our study found that BLJS generates higher peak ankle internal rotation moments than BRJS, which contributes to the risk of LAS injuries. Based on these effects, BLJS is particularly susceptible to LAS, especially under unexpected conditions, where the risk of injury is exacerbated.

### 4.4 Application significance

This study is the first to explore the influence of anticipated and movement factors on the lower limb biomechanics of two backhand-side SL movements. When athletes perform BRJS and BLJS in a non-anticipated environment, both movements show greater VILR and knee extension moments, which are correlated with ACL injury risk. We observed that under non-anticipated conditions, both movements exhibited a greater ROM in hip internal/external rotation and ankle inversion/eversion, serving as a lower limb self-protective mechanism that effectively reduces internal stress on the knee joint. However, the timing of this protective mechanism occurs after the vulnerable regions for ACL injury, suggesting that this non-anticipated protective mechanism may not reduce the risk of ACL injury (Krosshaug et al., 2006).

The two movements exhibited distinct landing biomechanical characteristics and ACL injury risk mechanisms. The main effect results indicate that BRJS demonstrates greater knee abduction angles and knee internal rotation moment, leading to a frontal plane ACL injury risk mechanism. Furthermore, the interaction effect shows that BRJS leads to a greater frontal plane COP displacement under non-anticipated conditions, which indirectly increases knee abduction and results in a higher ACL injury risk in non-anticipated environments. In the main effect comparison, BLJS exhibits a larger knee extension moment and smaller knee flexion and hip flexion angles, corresponding to a sagittal plane ACL injury risk mechanism. Additionally, compared to BRJS, BLJS shows a greater ankle internal rotation moment, which increases the risk of LAS. In the interaction effect, BLJS shows greater ankle inversion moments under non-anticipated conditions, further amplifying the LAS injury risk.

The results of this study highlight the potentially harmful impact of performing BRJS and BLJS on lower limb biomechanics when athletes are exposed to non-anticipated environments. These findings have important and direct implications for tactical design in badminton, as well as for badminton competitions and daily training. Training that focuses on central control mechanisms could significantly improve athletic response, especially in the inherently unpredictable environment of badminton (Borotikar et al., 2007). For example, incorporating direction-change tasks with varying speeds and directions, combined with visual and auditory feedback, could strengthen athletes’ decision-making responses under non-anticipated conditions, thereby reducing the risk of injury during training and competitions. Such training would improve athletes’ short-term decision-making and reaction abilities, while enhancing lower limb coordination and neuromuscular control, ultimately reducing the occurrence of sports-related injuries.

### 4.5 Limitations

This study has some limitations. First, the study relied on a laboratory environment, where visual stimuli were used to simulate unexpected conditions. These stimuli may not fully replicate the unexpected situations athletes encounter in real competitions, as they likely represent a simplified simulation. The movement of the opponent during a match is also a critical factor, not just the trajectory of the shuttlecock. Second, this study only included female badminton athletes ranked at level two or higher and lacked male participants. These athletes had extensive specialized training and considerable competitive experience. Compared to recreational athletes, the athletes included in this study had a distinct advantage in terms of sport-specific decision-making. Therefore, the findings of this study are applicable primarily to elite female badminton players and may not be directly generalized to other groups. Furthermore, gender differences in decision-making time and visual judgment suggest that the findings of this study are limited to female athletes (Ford et al., 2005; McLean et al., 2004). Finally, although *a priori* power analysis was conducted, the small sample size (n = 13) limits the ability to detect small effects. While the findings are valuable, it is important to note that the small sample size may affect the generalizability of the conclusions. Future studies may consider increasing the sample size or adopting experimental designs that more closely simulate real competition scenarios to enhance the broader applicability of the results and validate these findings.

## 5 Conclusion

This study demonstrates that executing BRJS and BLJS actions in non-anticipated environments results in greater VILR and knee extension moments. The main effect results indicate that compared to BLJS, BRJS shows greater knee abduction angles and knee internal rotation moment, and the interaction effect reveals that BRJS results in greater frontal plane COP displacement in non-anticipated conditions, which indirectly increases knee abduction and results in a higher frontal plane ACL injury risk. Additionally, in the main effect comparison, BLJS shows greater knee extension moments and smaller knee and hip flexion angles, which are linked to sagittal plane ACL injury mechanisms. Compared to BRJS, BLJS also shows greater ankle internal rotation moments, and the interaction effect indicates that in non-anticipated conditions, BLJS exhibits greater ankle inversion moments, which increases the LAS injury risk.

## Data Availability

The original contributions presented in the study are included in the article/Supplementary Material, further inquiries can be directed to the corresponding author.
