# Supplementary material for: Impact of unanticipated and backhand area smash landing on the lower limb biomechanics of female badminton players
Source: Front Bioeng Biotechnol. 2025 May 30;13:1609911. doi: 10.3389/fbioe.2025.1609911 (PMC12162505; doi:10.3389/fbioe.2025.1609911)
Supplement: Supplementary file 1 [file Table1.DOCX]

Supplementary Material

# Supplementary Table 1: Comparison of the effect sizes ($\boldsymbol{\eta}_{\boldsymbol{p}}^{\boldsymbol{2}}$) of biomechanical parameters across the combinations of BRJS and BLJS under anticipated and unanticipated conditions.

|  | | Effect Size ($\eta_{p}^{2}$) | | |
| --- | --- | --- | --- | --- |
|  |  | Anti | Movement | Interaction |
| COP (mm) | | | | |
| The Sagittal plane displacement | | 0.072 | 0.198 | 0.073 |
| The Front plane displacement | | 0.057 | 0.186 | 0.162 |
| ROM (°) | | | | |
| Ankle | Extension-Flexion | 0.041 | 0.302 | 0.049 |
|  | Eversion-Inversion | 0.166 | 0.008 | 0.01 |
|  | External-Internal Rot | 0.001 | 0.307 | 0.104 |
| Knee | Extension-Flexion | 0.008 | 0.075 | 0.004 |
|  | Abduction-Adduction | 0.021 | 0.037 | 0.006 |
|  | External-Internal Rot | 0.002 | 0.015 | 0.065 |
| Hip | Extension-Flexion | 0.003 | 0.5 | 0.006 |
|  | Abduction-Adduction | 0.11 | 0.035 | 0.044 |
|  | External-Internal Rot | 0.406 | 0.305 | 0.058 |
| Joint angle at IC (°) | | | | |
| Ankle | Dorsiflexion | 0.038 | 0.117 | 0.031 |
|  | Eversion | 0.118 | 0.061 | 0.004 |
|  | External Rot | 0.034 | 0.067 | 0.003 |
| Knee | Extension | 0.044 | 0.347 | 0.002 |
|  | Abduction | 0.023 | 0.182 | 0.004 |
|  | External Rot | 0.006 | 0.153 | 0.033 |
| Hip | Flexsion | 0.005 | 0.491 | 0.003 |
|  | Abduction | 0.034 | 0.133 | 0.232 |
|  | External Rot | 0.078 | 0.001 | 0.001 |
| loading rate | VALR | 0.149 | 0.004 | 0.006 |
|  | VILR | 0.314 | 0.053 | 0.002 |
| Joint moment at IC (Nm/kg) | | | | |
| Ankle | Dorsiflexion | 0.006 | 0.068 | 0.026 |
|  | Eversion | 0.001 | 0.066 | 0.091 |
|  | External Rot | 0.006 | 0.344 | 0.029 |
| Knee | Extension | 0.231 | 0.161 | 0.035 |
|  | Abduction | 0.078 | 0.248 | 0.007 |
|  | External Rot | 0.009 | 0.187 | 0.008 |
| Hip | Flexsion | 0.045 | 0.238 | 0.015 |
|  | Abduction | 0.218 | 0.103 | 0.065 |
|  | External Rot | 0.006 | 0.342 | 0.015 |
| Joint moment at 1^st^ vGRF phase | | | | |
| Knee | Extension | 0.284 | 0.074 | 0.071 |
|  | Abduction | <0.001 | 0.364 | 0.044 |
|  | External Rot | 0.047 | 0.124 | 0.28 |
| Peak ankle moment (Nm/kg) | | | | |
| Dorsiflexion | | 0.049 | 0.001 | 0.06 |
| Eversion | | 0.103 | 0.005 | 0.212 |
| External Rot | | 0.053 | 0.279 | 0.071 |

# Supplementary Table 2: Summary of SPM significance results

|  | | Anti | | Movement | | Interaction | |
| --- | --- | --- | --- | --- | --- | --- | --- |
|  |  | p-value | Cluster location | p-value | Cluster location | p-value | Cluster location |
| Kinematics | | | | | | | |
| Ankle | Extension-Flexion | N.S |  | p=0.024 | 34.1%-67.3% | N.S |  |
|  | Eversion-Inversion | N.S |  | N.S |  | N.S |  |
|  | External-Internal Rot | N.S |  | N.S |  | N.S |  |
| Knee | Extension-Flexion | N.S |  | p=0.05 | 0%-2.5% | N.S |  |
|  | Abduction-Adduction | N.S |  | p=0.05 | 0%-4.9% | N.S |  |
|  | External-Internal Rot | N.S |  | p=0.048 | 0%-8.8% | N.S |  |
| Hip | Extension-Flexion | N.S |  | p=0.019 | 0%-42.4% | N.S |  |
|  | Abduction-Adduction | N.S |  | N.S |  | P<0.001 | 0%-100% |
|  | External-Internal Rot | N.S |  | N.S |  | N.S |  |
| Kinetics | | | | | | | |
| Ankle | Extension-Flexion | N.S |  | N.S |  | N.S |  |
|  | Eversion-Inversion | N.S |  | N.S |  | N.S |  |
|  | External-Internal Rot | N.S |  | P=0.003 | 0%-64.2% | N.S |  |
| Knee | Extension-Flexion | p<0.001 | 0%-86.6% | p=0.044 | 86.4%-100% | N.S |  |
|  | Abduction-Adduction | N.S |  | p=0.014 | 0%-40.9% | N.S |  |
|  | External-Internal Rot | N.S |  | p=0.03, p=0.048 | 0%-32.2%, 91%-100% | N.S |  |
| Hip | Extension-Flexion | N.S |  | p=0.035 | 0%-26.6% | N.S |  |
|  | Abduction-Adduction | p=0.004 | 9.8%-87.5% | p=0.024, p=0.036; | 10.2%-51.5%, 72.3-100% | N.S |  |
|  | External-Internal Rot | N.S |  | p=0.045 | 0%-10.6% | N.S |  |
| Vertical ground reaction force | | |  | p=0.037 | 57.5%-60.4% | p=0.003 | 53.1%-62.1% |

N.S: no significant difference.
